# RaptGen-Assisted Generation of an RNA/DNA Hybrid Aptamer against SARS-CoV-2 Spike Protein

**DOI:** 10.1021/acs.biochem.3c00596

**Published:** 2024-03-08

**Authors:** Tatsuo Adachi, Shigetaka Nakamura, Akiya Michishita, Daiki Kawahara, Mizuki Yamamoto, Michiaki Hamada, Yoshikazu Nakamura

**Affiliations:** † RIBOMIC Inc., 3-16-13 Shirokanedai, Minato-ku, Tokyo 108-0071, Japan; ‡ Graduate School of Advanced Science and Engineering, Waseda University, 3-4-1, Okubo Shinjuku-ku, Tokyo 169-8555, Japan; § Computational Bio Big-Data Open Innovation Laboratory (CBBD-OIL), National Institute of Advanced Industrial Science and Technology (AIST), 3-4-1, Okubo Shinjuku-ku, Tokyo 169-8555, Japan; ∥ Research Center for Asian Infectious Diseases, The Institute of Medical Science, The University of Tokyo, 4-6-1 Shirokanedai, Minato-ku, Tokyo 108-8639, Japan; ⊥ The Institute of Medical Science, The University of Tokyo, 4-6-1 Shirokanedai, Minato-ku, Tokyo 108-8639, Japan

## Abstract

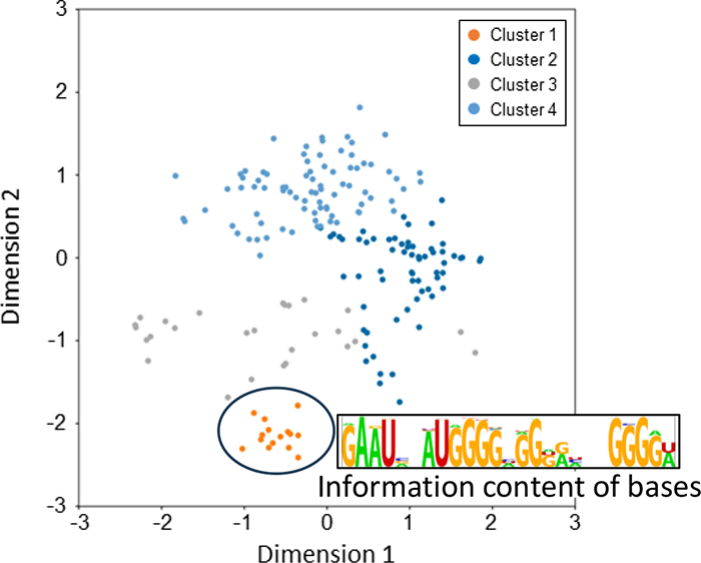

Optimization of aptamers in length and chemistry is crucial for industrial applications. Here, we developed aptamers against the SARS-CoV-2 spike protein and achieved optimization with a deep-learning-based algorithm, RaptGen. We conducted a primer-less SELEX against the receptor binding domain (RBD) of the spike with an RNA/DNA hybrid library, and the resulting sequences were subjected to RaptGen analysis. Based on the sequence profiling by RaptGen, a short truncation aptamer of 26 nucleotides was obtained and further optimized by a chemical modification of relevant nucleotides. The resulting aptamer is bound to RBD not only of SARS-CoV-2 wildtype but also of its variants, SARS-CoV-1, and Middle East respiratory syndrome coronavirus (MERS-CoV). We concluded that the RaptGen-assisted discovery is efficient for developing optimized aptamers.

Aptamers are single-stranded oligonucleotides that bind to specific target molecules. Aptamers have been used in various fields such as medicines, diagnostics, and separation agents because of their high affinity and specificity toward targets.^1^ Aptamers are generated by an in vitro molecular evolution method known as systematic evolution of ligands by exponential enrichment (SELEX).^2,3^ After candidate identification, the chemical properties of aptamers should be optimized for industrial use. First, the aptamer length should be as short as possible. Aptamer truncation will reduce the cost of manufacturing and facilitate material quality assurance. Second, nucleotide modifications should be included for chemical stability. Nuclease resistance is required especially for therapeutic applications.^1^ Ribose modifications, such as 2′-fluoro-ribose and 2′-*O*-methyl-ribose are widely used to confer nuclease-resistance. In general, point-by-point modification is needed to ensure the activity of the aptamers. Sequence truncation is again beneficial in chemical optimization processes because it will reduce the possible number of combinations of substitutions. Several customized SELEX methods have been developed to generate short aptamers.^4−6^ Fixed sequences for polymerase chain reaction (PCR) amplification are removed during affinity selection in the primer-less SELEX.^4,5^ Collectively, selection strategies for short candidates are critical in aptamer development.

Computational approaches have been developed for efficient aptamer discovery. Since the recent development of next-generation sequencing provides vast sequence information, bioinformatical approaches are receiving attention for analyzing SELEX data. They are used to estimate motif information in the candidate sequences for instance.^7,8^ We recently developed a deep-learning-based tool, RaptGen.^9^ RaptGen embeds SELEX data into a low-dimension space where sequence features are distributed in a motif-dependent manner.^9^ RaptGen is also able to generate sequence profiles from the latent space based on cluster information.^9^ Therefore, RaptGen could propose seed aptamers harboring motif information. Since the current version of RaptGen deals with a randomized region of the SELEX library independently of fixed primer regions, RaptGen could be applicable to primer-less SELEX data. Hence, we thought that selection strategies using RaptGen are worth considering for short aptamer discovery.

Recent emergence of COVID-19 and the causal virus, SARS-CoV-2, have had a serious impact on human society. So far, several anti-SARS-CoV-2 aptamers are reported.^10−17^ Most of the previous SELEX targets the spike protein, especially the receptor binding domain (RBD), which is used for virus-host interaction.^18^ Using as diagnostic reagents is one of the possible applications of anti-SARS-CoV-2 aptamers. For example, Yang et al. produced DNA aptamers and they proposed a lateral flow detection system.^11^ Another challenging application of anti-SARS-CoV-2 aptamers is developing as antiviral agents. SARS-CoV-2 viral entry is thought to be initiated by RBD binding toward the host receptor, such as ACE2.^19^ Thus, aptamers inhibiting the RBD-ACE2 interaction could reduce viral entry into the cells. Liu and co-workers demonstrated that DNA aptamers prevent RBD binding from ACE2.^10^ Hence, these aptamers are potentially used as antiviral agents. Some effort for exploring nucleotide combinations has been made to discover new aptamers.^16,17^ Minagawa et al. reported a novel anti-SARS-CoV-2 aptamer using base-appended-base.^17^ There remains a variety of nucleotide combinations to be tested. Collectively, anti-SARS-CoV-2 aptamers and their applications have attracted growing interest.

In this study, we generated anti-SARS-CoV-2 RBD aptamers using an RNA/DNA hybrid substrate. To minimize the length of the aptamer, we exploited a primer-less SELEX strategy and RaptGen analysis. We further included chemical modifications into the aptamer and evaluated binding activity of these aptamers to SARS-CoV-2 variants, SARS-CoV-1, and MERS.

## Materials and Methods

### General

Aptamers in the first screening and truncation step were produced by in vitro transcription using T7 RNA polymerase harboring the Y639F mutation. All substrates were purchased from GeneAct (Fukuoka, Japan). All chemically modified aptamers were synthesized at GeneDesign (Osaka, Japan) and Hokkaido System Science (Hokkaido, Japan). All evaluated sequences are listed in Table S1. Biotinylated aptamers were synthesized using 3′-Biotin-TEG CPG.

### SELEX Experiment

SELEX was carried out by using a method of primer-less SELEX with some modifications as described previously.^20^ A single-stranded DNA (ssDNA) library, 5′-TCGAG-25N-ACCCTATAGTGAGTCGTATTA-3′, was used as the template. Here, 25N represents the 25-nt random sequence, and the underlined sequence indicates the complementary sequence of the T7 promoter. Using this library, we produce the 34-nt aptamer containing 25-nt random region, GGGT at 5′-end, and CTCGA at 3′-end for ligation reaction. First, the ssDNA template was hybridized with the forward primer, 5′-TGGAGCGAACTAGACTAATACGACTCACTATAGGGT-3′, and blunt end dsDNA was produced by DNA polymerase. The random 25N RNA/DNA hybrid library was then transcribed using T7 RNA polymerase harboring Y639F mutation, nucleotides of 2′-OH-GTP, 2′-OH-ATP, 2′-deoxy-CTP, 2′-deoxy-TTP and 10 molar excess condition of GMP relative to GTP. GMP was added to generate a monophosphorylated 5′ terminal, which is essential for following the ligation reaction. The resulting oligonucleotide pool was used for binding selection to the RBD protein (40592-V08H, Sino Biological), which was immobilized to NHS-activated Sepharose beads (17-0906-01, Cytiva). The first round of selection was performed with 2 μg of RBD protein and 10 μg of the input RNA/DNA hybrid library, which consisted of roughly 10^14^ unique sequences. Protein and library incubated for 30 min in 50 μL of buffer consisting of 145 mM NaCl, 5.4 mM KCl, 0.8 mM MgCl_2_, 1.8 mM CaCl_2_, 0.05% Tween20 and 20 mM Tris–HCl (pH 7.6). After incubation, the beads were washed three times with the same buffer, and the RNA/DNA molecules bound to RBD were eluted with 6 M urea. To increase the stringency of the selection, in rounds 4, 5, and 6, a high salt concentration buffer containing 295 mM NaCl was used for washing. For the subsequent round of selection and amplification, the T7 promoter sequence (5′-TAATACGACTCACTATA-3′) was ligated to the 5′ terminus of the selected RNA/DNA sequences in the presence of the forward bridge sequence (5′-ACCCTATAGTGAGTCGTATTA-NH_2_-3′), and the 3′ terminus of the selected RNA/DNA sequences was ligated to the reverse adaptor sequence (5′-p-GAATAAGCAAAAGATAT-NH_2_-3′) in the presence of the reverse primer sequence (5′-ATATCTTTTGCTTATTCTCGAG-3′) by using T4 RNA ligase 2 (M0239, New England Biolabs), and reverse-transcribed by SuperScript IV (18090050, Thermo Fisher Scientific). After PCR amplification, the dsDNA was digested by the *Xho*I restriction enzyme (R0146S, NEB) and used as the library for the next round. The nucleotide pools were analyzed with an Ion PGM instrument and Ion PGM Hi-Q View Sequencing Kit (A30044, Thermo Fisher Scientific).

### RaptGen Analysis

All sequences with exact matching adapters and sequence design lengths and more than 3 read counts were selected, and unique random regions of these sequences were used as input for RaptGen. The embedding dimension was specified to be two. The model showing the lowest test loss was selected from 30 trained models to use analysis. Other parameters were set to default values.^9^

Candidate sequences were selected from the sequence data by following the procedure. First, the latent embeddings were separated into several Gaussian distributions based on a Gaussian mixture model. The mixture number was determined by the Bayesian information criterion.^21^ The most probable sequences were reconstituted from each distribution center according to the previous report.^9^ Among sequences having edit distance from reconstituted sequences less than 10, the most frequently appearing sequence was selected from the deep sequencing data. Nonbinding sequences and their analogues were removed in the case of the second trial of RaptGen analysis. The remaining sequencing data were used as input for RaptGen. Candidate sequences were selected using the same procedures described above.

### Surface Plasmon Resonance Assay

The surface plasmon resonance (SPR) assays were performed using a Biacore T200 instrument (Cytiva) as described previously with slight modifications.^22^ To analyze the deletion mutant series, aptamers were synthesized with 16-mer polyA-tails as follows: 5′-GGGT–(variable sequence)-CTCGA-(polyA)-3′ and transcribed in vitro using the same method for library preparation. The running buffer consisting of 145 mM NaCl, 50 mM KCl, 0.8 mM MgCl_2_, 1.8 mM CaCl_2_, 0.05% Tween20, and 20 mM Tris–HCl (pH 7.6) were used for all SPR experiments. A 5′-biotinylated dT16 oligomer was immobilized to both active and reference flow cells of the streptavidin sensor chip (BR100531, Cytiva). The poly(A)-tailed RNA was captured in the active flow cell by complementary hybridization at a concentration of 200 nM and a flow rate of 20 μL/min with an association time of 60 s. The proteins were injected into the flow cells of the sensor chip at a concentration of 100 nM and a flow rate of 30 μL/min, with an association time of 60 s. The sensor chip was regenerated by injecting 6 M urea to remove the bound aptamers. Data were obtained by subtracting the reference flow cell data from the active flow cell data. The maximum response after injection was used for analysis.

For evaluating chemical modification derivatives, spike protein subunit S1 (40591-V08H, Sino Biological) was immobilized on active flow cells of the CM5 sensor chip (BR100531, Cytiva) by an amine coupling kit (BR-1000-50, Cytiva) according to the manufacturer’s instruction. The target level was at 3000–3500 RU. For binding analysis, 200 nM aptamers were injected at a flow rate of 30 μL/min, with an association time of 60 s. To regenerate the sensor chip, bound aptamers were removed by injecting 2 M NaCl and 1 mM Glycine (pH 2.0) for 60 s.

For the *K*_D_ calculation, a biotinylated aptamer was synthesized and immobilized on the active flow cells of the streptavidin sensor chip (BR100531, Cytiva). Serial dilutions of recombinant proteins, RBD-wildtype (40592-V08H, Sino Biological), spike trimer (SPN-C52H9, ACROBiosystems), RBD-N501Y (SPD-C52HN, ACROBiosystems), RBD-E484 K (SRD-C52H3, ACROBiosystems), RBD-N501Y/E484 K/K417N (SPD-C52HP, ACROBiosystems), RBD-Omicron (SPD-C522E, ACROBiosystems), RBD-SARS-CoV-1 (SPD-S52H6, ACROBiosystems) and RBD-MERS (SPD-M52H6, ACROBiosystems), at final concentrations of 0–25 nM were injected. The flow rate was maintained at 30 μL/min during the whole process. The association and dissociation time was kept at 60 and 300 s, respectively. Regeneration was carried out with 6 M Guanidine-HCl for 30 s. Data analysis was carried out using Biacore T200 evaluation software and fitted to the 1:1 binding model.

### Flow Cytometry Analysis

A plasmid coding Spike protein (NCBI Reference Sequence: YP_009724390.1) was transfected to HEK293FT. About 0.2 million transfected cells were resuspended in binding buffer (145 mM NaCl, 50 mM KCl, 0.8 mM MgCl_2_, 1.8 mM CaCl_2_, 20 mM Tris–HCl (pH 7.6)) supplemented with 1 mg/mL tRNA and 1 mg/mL Salmon Sperm DNA. For refolding aptamers, aptamers with FAM labels were diluted in buffer and thermally equilibrated by heating to 85 °C for 3 min, then left to cool at room temperature for 10 min. Subsequently, cells were stained with 1 μM labeled aptamers for 30 min at 37 °C. Cells were washed with binding buffer 3 times and resuspended in binding buffer. Samples were analyzed by flow cytometry (FACSMelody, BD). For antibody staining, transfected cells were resuspended in D-PBS (−) and then incubated with 1:75 anti-SARS-CoV-2 (2019-nCoV) spike neutralizing monoclonal mouse antibody (40591-MM43, Sino Biological) for 30 min at 4 °C. Cells were then washed with D-PBS 3 times and a secondary antibody mouse IgG1-PE was added at a dilution of 1/200 before 30 min incubation at 4 °C. Cells were washed with D-PBS (−) 3 times for FACS analysis.

## Results

### Identification of an RNA/DNA Hybrid Aptamer against SARS-CoV-2

**Figure 1:**
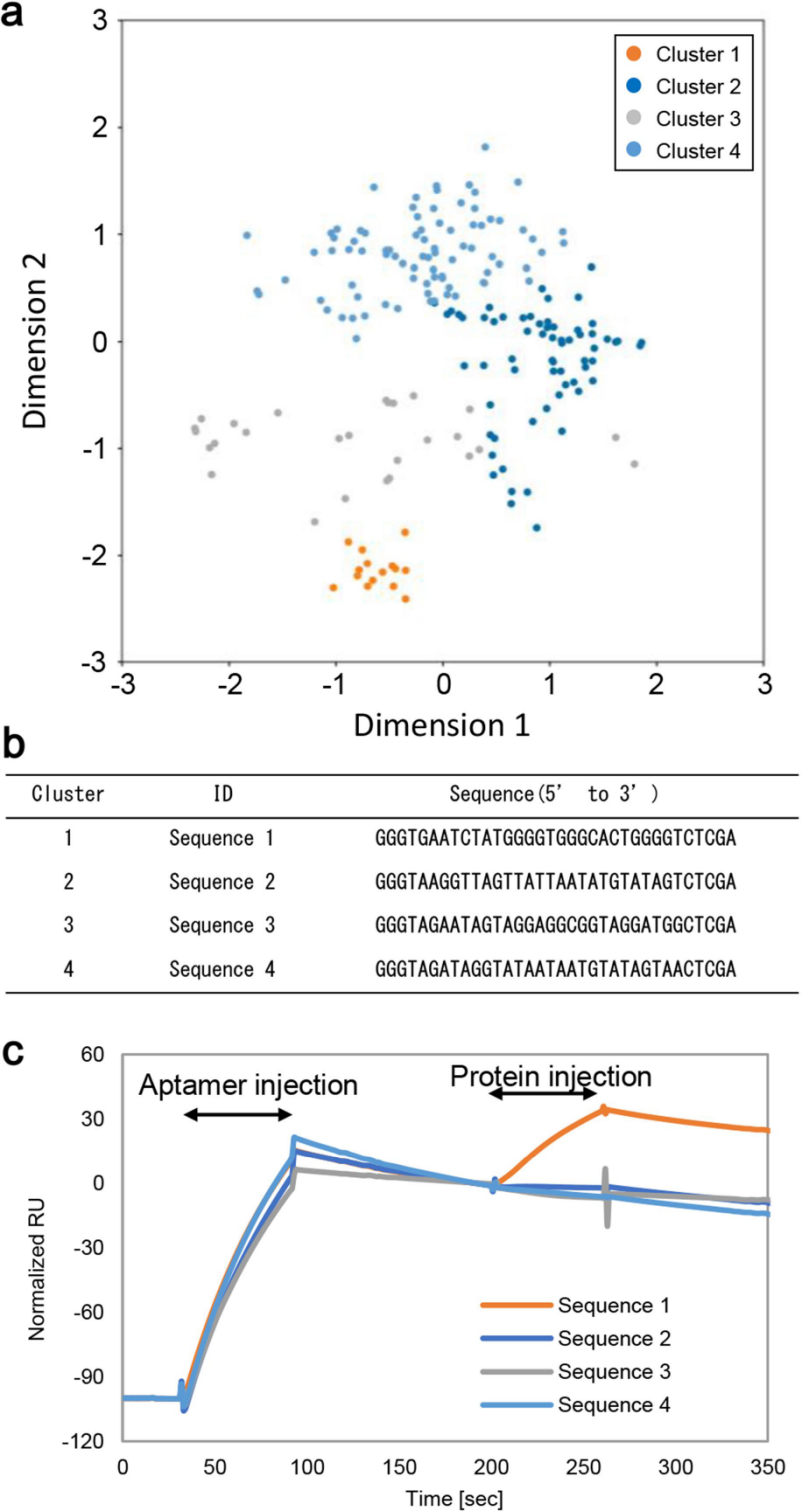
Candidate discovery using RaptGen. (a) Preprocessed sequencing data was subjected to RaptGen. After creating a latent space, representative sequences were selected as aptamer candidates. The plots indicate individual sequences in the sequencing data. The plots are indicated in the same color as the representative sequence. (b) Candidate sequences were listed in the table. A, G: RNA, C, T: DNA (c) Binding activity of candidate sequences was assessed by the SPR experiment. PolyA-tailed aptamers were generated by in vitro transcription. After aptamer immobilization on a sensor chip, 100 nM RBD protein was injected.

We first conducted a SELEX against a receptor-binding domain (RBD) of SARS-CoV-2. We constructed an RNA/DNA hybrid oligonucleotide library, which is composed of the purine ribonucleotides and the pyrimidine deoxy ribonucleotides. Since RNase A degrades RNA at pyrimidine ribonucleotides,^23^ replacing them for deoxyribonucleotides is beneficial to discover nuclease-resistant aptamers. The Y639F T7 RNA polymerase was reported to incorporate deoxyribonucleotide.^24^ We therefore chose this nucleotide and polymerase combination. To obtain short aptamer candidates, we adopted a primer-less SELEX strategy. After six rounds of in vitro selection, the sequencing data was subjected to RaptGen software. We created a two-dimension latent space according to the previous report^9^ (Figure S1a). We found that most of the sequence profiles were less informative because the information content of bases was low (Figure S2a). We generated aptamer candidates from the five clusters and tested the binding activity (Figure S1b). We found that no sequence displayed RBD binding activity (Figure S1c). Then we attempted to exclude nonbinding sequences to focus on the minor population. The nonbinding candidates and their analogues (i.e., edit distance less than 10) were removed from the original sequencing data and reanalyzed by RaptGen. The latent space according to the second learning was created (Figures 1a and S2b). Although the base proportion was not dramatically changed during the SELEX (Figure S3), we noticed that G-rich sequence populations emerged (Figure S2b). Four candidates representing clusters were selected (Figure 1a,b). We found that Sequence 1 is bound to the RBD (Figure 1c). The Sequence 1 is hereafter called SPA1. Next, to conduct sequence truncation, we designed mutant aptamers harboring a single nucleotide deletion and produced the aptamers by in vitro transcription. All truncated aptamers were analyzed by SPR assay to assess the binding activities toward RBD (Table 1, Figure S4). In the case of SAP1-T01 to T03, relative binding activity is higher than that of SPA1. These results indicated that a few bases around these 3′ ends are not involved in binding to the RBD. On the other hand, in the case of SPA1-T04 and T06, these aptamers have no binding activity to RBD protein. Therefore, we concluded that the deleted bases in these sequences are essential for binding to RBD. Deletion of guanosine located in guanosine repeats led to loss of aptamer activity. Thus, we supposed that SPA1 forms a G-quadruplex structure. There are some cases that non- guanosine-deletion diminished SPA1 binding as shown in SPA1-T05 and SPA1-T07, indicating SPA1 has more binding activity than a simple G-quartet oligonucleotides. Noteworthy, indispensable nucleotides were well consistent with the information content of bases proposed by RaptGen (Table 1). These results suggest that RaptGen is applicable to primer-less SELEX data and can be used for motif estimation. A truncated version SPA1 was composed of 26 nt (referred to as SPA1-T16). We used this short-length aptamer for the following experiment.

**Table 1:**
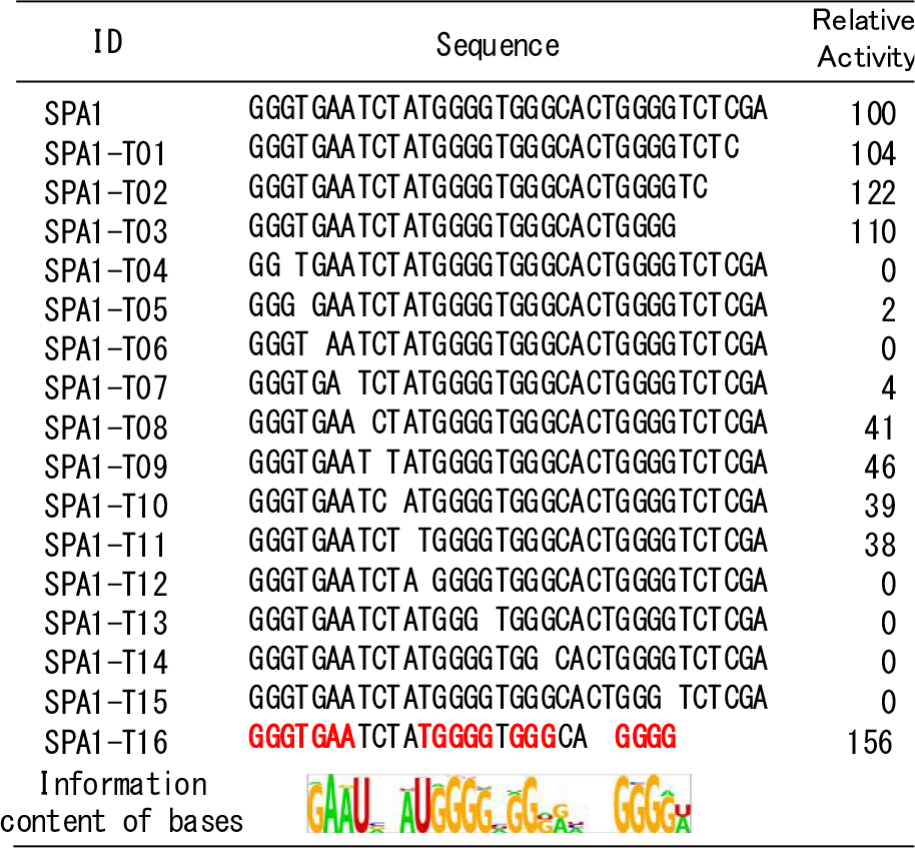
Binding Activity of Truncated Aptamers against the RBD; A, G: RNA, C, T: DNA

### Chemical Synthesis and Modification of SPA1

We next optimized chemical modifications into SPA1-T16. To achieve position-specific modifications, several modified aptamers were chemically synthesized and assessed their activities (Table 2, Figure S5). The original SPA1-T16 produced by in vitro transcription is supposed to be phosphorylated at 5′ terminal because we had used GMP primer during the primer-less SELEX. We, therefore, evaluated unphosphorylated SPA1-T16. Unexpectedly, the binding activity of the unphosphorylated SPA1-M02 was greatly reduced. Hence, this phosphate group is indispensable for binding. We next introduced 2′-*O*-methyl modification, which is widely used for oligonucleotide medicines. We first modified natural RNA in the middle of the sequence, and then we introduced phosphorothioate linkages at both ends. Some combinations of 2′-*O*-methyl modifications were permissible (Table 2). We also found that a single substitution introduced in SPA1-M09 could diminish the SPA1 activity. We further implemented phosphorothioate modification at both the ends of the oligonucleotides and the remaining unmodified purines. We revealed that terminal phosphorothioate strengthened the binding, as shown in SPA1-M12. The final aptamer, SPA1-M13, no longer contains natural ribonucleotides.

**Table 2:**
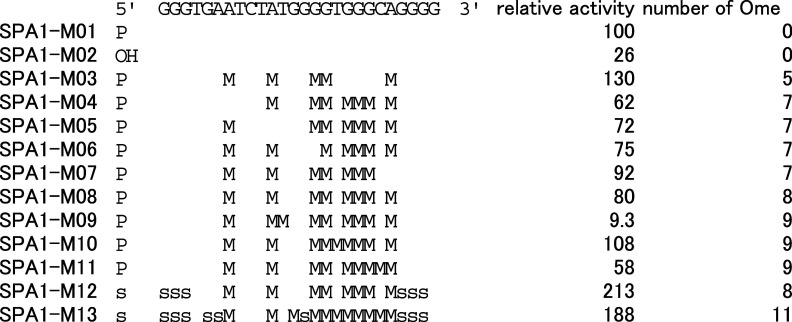
Binding Activity of Chemically Modified Aptamer against S1 Protein^a^

a P: Phosphate, OH: Hydroxy, M: 2′-*O*-methyl, s: Phosphorothioate.

**Figure 2:**
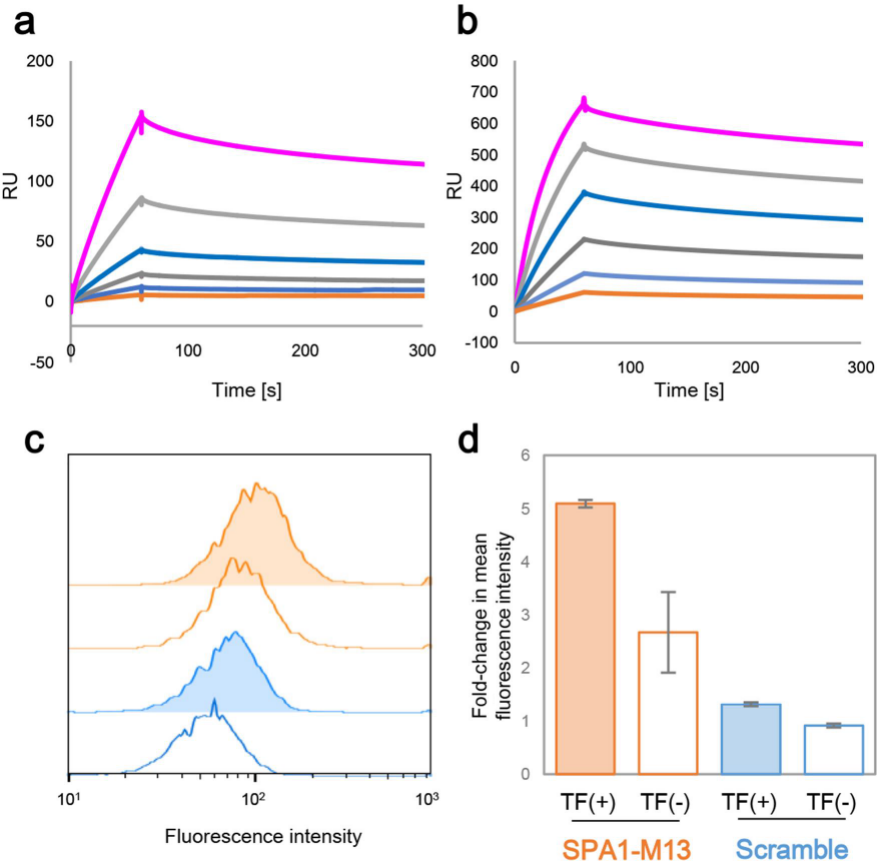
Binding profile of SPA1-M13 to the (a) RBD or (b) trimer of the spike protein. Biotinylated SPA1-M13 was immobilized on a streptavidin sensor chip. *K*_D_ measurements were performed by SPR using different concentrations (25.0 12.5, 6.25, 3.12, 1.56, and 0.78 nM) of target protein. (c) HEK293FT cells were transfected with a plasmid coding Spike protein. Binding of FAM-labeled SPA1-M13 aptamer toward transfected (TF (+); orange) or nontransfected (TF (−); blue) cells were assessed by flow cytometry. Scramble sequence was used as a negative control. (d) Fold-change in mean fluorescent intensity (MFI) compared to unstained cells were shown. Error bars represent standard deviation of three independent experiments.

In order to evaluate the properties of this SPA1-M13 aptamer, we first measured the *K*_D_ value using the SPR assay. We additionally evaluated SPA1-M13 binding to a trimer form of the Spike protein, which is thought to be displayed on the SARS-CoV-2 surface. SPA1-M13 showed nanomolar level binding affinity at *K*_D_ of 2.8 nM and 0.39 nM toward RBD and spike protein trimer, respectively (Figure 2a,b). We further attempted to assess the binding of SPA1-M13 to the Spike protein on the cellular membrane by using flow cytometry. We transfected a Spike protein expression plasmid into HEK293FT cells. We demonstrated that a FAM labeled SPA1-M13 had a higher staining intensity toward transfected cells than nontransfected cells (Figure 2c,d). We noticed spike protein-independent binding between SPA1-M13 and nontransfected cells. We also showed that SPA1-M13 had a superior binding compared with a scramble sequence of SPA1-M13 (Figure 2c,d). We therefore thought that SPA1-M13 could recognize the spike protein on the viral particles. Collectively, SPA1-M13 is the most shortened and chemically modified aptamer for SARS-CoV-2 up to the present.

### SPA1 Binding to SARS-CoV-2 Variants

**Figure 3:**
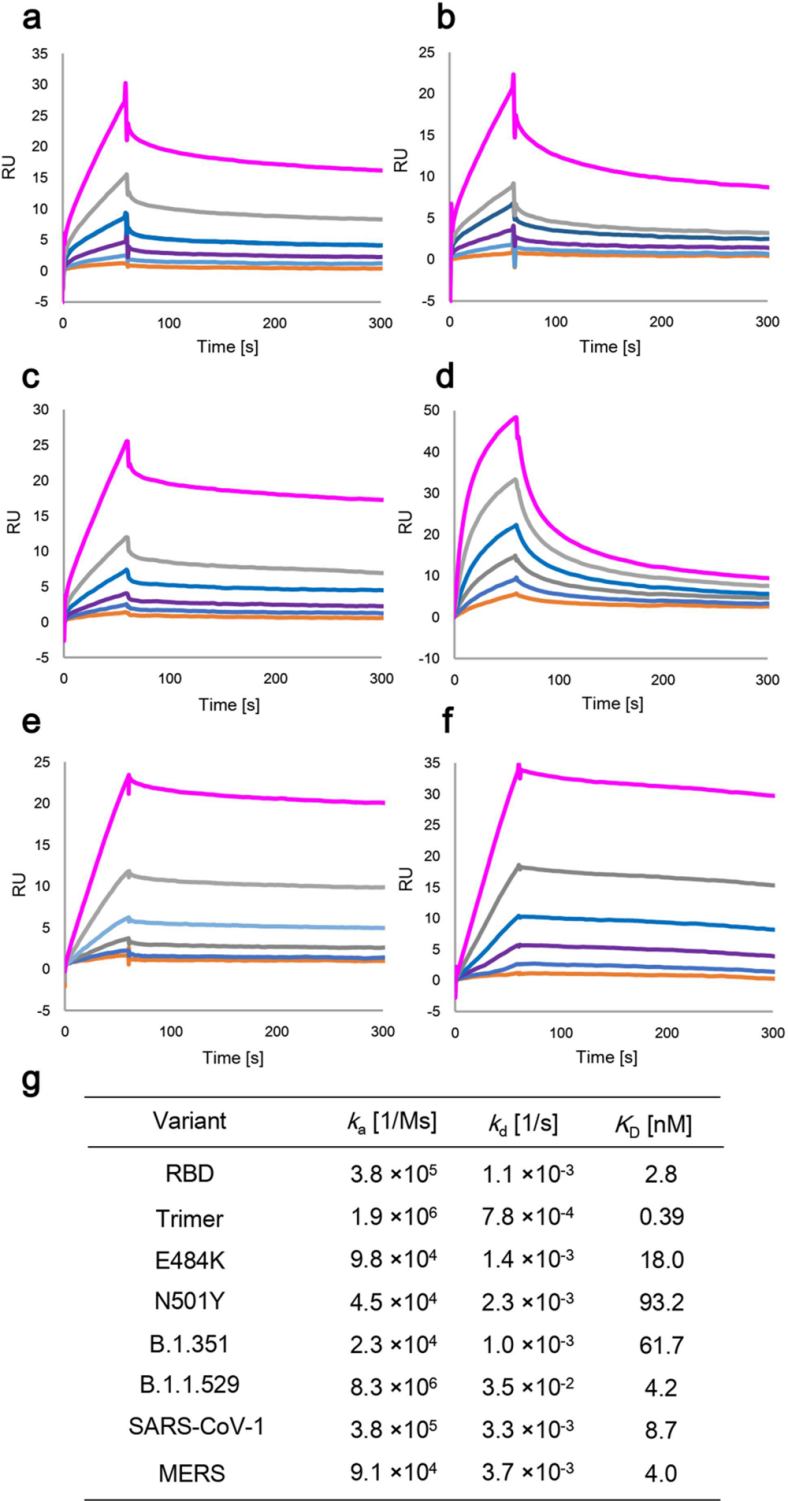
Binding profiles of SPA1 aptamer to SARS-CoV-2 variants by SPR. Biotinylated SPA1-M13 aptamer was immobilized on a streptavidin chip, and different concentrations (25.0 12.50, 6.25, 3.12, 1.56, and 0.78 nM) of recombinant proteins were injected. SPR sensorgrams of the affinity of SPA1-M13 aptamer to (a) E484, (b) N501Y, (c) B.1.351, (d) B.1.1.529, (e) SARS-CoV-1, and (f) MERS were shown. Each parameter of binding kinetics between SPA1-M13 and RBD variant were summarized in (g).

We evaluated the binding activity of SPA1-M13 toward RBD proteins from SARS-CoV-2 variants. A SARS-CoV-2 variant carrying the amino acid substitution N501Y in the RBD is one of the most prevalent mutations found in the COVID-19 cases. Another mutation, the E484K substitution alone, has been shown to confer resistance to several monoclonal antibodies. E484 exists in the surface of the ACE2 binding region and therefore is an important epitope for neutralizing antibody. We assessed the binding activity of chemically modified SPA1-M13 against RBD protein carrying E484K, N501Y, or triple substitutions (N501Y/E484K/K417N). SPA1-M13 bound to E484K, N501Y, and triple substitutions variant with *K*_D_ at 18.0, 93.2, and 61.7 nM, respectively (Figure 3a–c). The binding affinity of SPA1-M13 and these mutant proteins was lower level than that of wild-type RBD, but it can bind to mutant proteins. We also assessed binding against RBD of the B.1.1.529 (Omicron) variant, which is recently considered as a variant of concern. SPA1-M13 also bound to the B.1.1.529 variant with a similar affinity of the wildtype (*K*_D_ = 4.2 nM). However, we found that the dissociation rate was increased (Figure 3d). Further study would reveal that which substitution affects the interaction between SPA1 and RBD. There have been other kinds of clinically important coronaviruses, such as SARS-CoV-1 and MERS. They also use spike protein and previous studies reported that some epitopes are conserved among them and SARS-CoV-2. We tested whether SPA1-M13 has affinity toward RBD of SARS-CoV-1 and MERS. As a result, SPA1 also bound RBD of SARS-CoV-1 and MERS with *K*_D_ at 8.7 and 4.0 nM, respectively (Figure 3e,f). These results suggest that SPA1-M13 has a broad range binding spectrum against coronavirus.

## Discussion

In this study, we performed a primer-less aptamer discovery using RaptGen and developed a novel anti-SARS-CoV-2 nucleic acid aptamer, SPA1. Modified SPA1 is bound to SARS-CoV-2 and its variants. SPA1 was heavily modified compared to other previously identified aptamers. To our knowledge, this is the first study describing a deep-learning-assisted primerless aptamer discovery. We concluded that the RaptGen-based strategy accelerates aptamer development.

The mode of action of therapeutic antibodies is mainly to block the entry of SARS-CoV-2 into the endothelial cells. Previous studies reported that the inhibitory activity of antibodies is dependent on their epitope. An antibody CR3022 binds to an epitope conserved between SARS-CoV-1 and SARS-CoV-2.^25^ Structural analysis revealed that the CR3022 epitope exists at different sites of receptor binding motif and would not clash with ACE2. In the present study, we showed that SPA1 is bound to several kinds of coronavirus RBD including SARS-CoV-1 and MERS. Hence, we supposed that the SPA1 binding region is unrelated to the receptor-binding surface like CR3022. This would be beneficial to develop a broad-spectrum inhibitor for coronavirus.

## Supporting Information Available

Sequence list; latent spaces; SPR sensorgrams; base proportions; and histograms (PDF)

## Accession Codes

RBD-wildtype (40592-V08H, Sino Biological): YP_009724390.1. spike trimer (SPN-C52H9, ACROBiosystems): QHD43416.1. RBD-N501Y (SPD-C52HN, ACROBiosystems): QHD43416.1 (N501Y). RBD-E484 K (SRD-C52H3, ACROBio-systems): QHD43416.1 (E484 K). RBD-N501Y/E484K/K417N (SPD-C52HP, ACRO-Biosystems): QHD43416.1 (K417N, E484K, N501Y). RBD-Omicron (SPD-C522E, ACROBiosys-tems): QHD43416.1 (G339D, S371L, S373P, S375F, K417N, N440K, G446S, S477N, T478K, E484A, Q493R, G496S, Q498R, N501Y, Y505H). RBD-SARS-CoV-1 (SPD-S52H6, ACROBiosystems): AAP13567.1. RBD-MERS (SPD-M52H6, ACROBiosystems): K0BRG7-1. surface glycoprotein [Severe acute respiratory syndrome coronavirus 2]: YP_009724390.1
