# Supplementary material for: RaptGen-Assisted Generation of an RNA/DNA Hybrid Aptamer against SARS-CoV-2 Spike Protein
Source: Biochemistry. 2024 Mar 8;63(7):906–12. doi: 10.1021/acs.biochem.3c00596 (PMC10993888; doi:10.1021/acs.biochem.3c00596)
Supplement: Supplementary file 1 — bi3c00596_si_001.pdf [file bi3c00596_si_001.pdf]

Title

RaptGen-assisted generation of an RNA/DNA hybrid aptamer against SARS-CoV-2 spike protein.

AUTHOR NAMES

Tatsuo Adachi[1]*, Shigetaka Nakamura[1], Akiya Michishita[2,3], Daiki Kawahara[1], Mizuki Yamamoto[4], Michiaki Hamada[2,3], Yoshikazu Nakamura[1,5]

AUTHOR ADDRESS

1. RIBOMIC Inc., 3-16-13 Shirokanedai, Minato-ku, Tokyo 108-0071, Japan
2. Graduate School of Advanced Science and Engineering, Waseda University, 3-4-1, Okubo Shinjuku-ku, Tokyo 169-8555, Japan.
3. Computational Bio Big-Data Open Innovation Laboratory (CBBD-OIL), National Institute of Advanced Industrial Science and Technology (AIST), 3-4-1, Okubo Shinjuku-ku, Tokyo 169-8555, Japan.
4. Research Center for Asian Infectious Diseases, The Institute of Medical Science, The University of Tokyo, 4-6-1 Shirokanedai, Minato-ku, Tokyo 108-8639, Japan.
5. The institute of Medical Science, The University of Tokyo, 4-6-1 Shirokanedai, Minato-ku, Tokyo 108-8639, Japan

E-mail

t.adachi@ribomic.com

Table S1. The sequence SPA1 aptamer series.

Sequence 1 to Sequence S5: Candidate sequences from RaptGen prediction.

SPA1-T01 to SPA1-T16; Truncated versions of Sequence 1.

SPA1-M01 to SPA1-M13: Chemically modified versions of SPA1-T16.

ID	Sequence (5' to 3')
Sequence 1	pGGGtGAAtctAtGGGGtGGGcActGGGGtctcGA-AAAAAAAAAAAAAAAA
Sequence 2	pGGGtAAGGttAGttAttAAtAtGtAtAGtctcGA-AAAAAAAAAAAAAAAA
Sequence 3	pGGGtAGAAtAGtAGGAGGcGGtAGGAtGGctcGA-AAAAAAAAAAAAAAAA
Sequence 4	pGGGtAGAtAGGtAtAAtAAtGtAtAGtAActcGA-AAAAAAAAAAAAAAAA
Sequence S1	pGGGtAGtAAtAAtAtAGtAtAtAGcGGGtctcGA-AAAAAAAAAAAAAAAA
Sequence S2	pGGGtAGtAGtAttGtAttAGtGtAtAtAtctcGA-AAAAAAAAAAAAAAAA
Sequence S3	pGGGtAtAGAAcAAttGtttAAtAAtGAAActcGA-AAAAAAAAAAAAAAAA
Sequence S4	pGGGtAGtAGtAGttttAtGtAtAGtAtAtctcGA-AAAAAAAAAAAAAAAA
Sequence S5	pGGGtAtAGAAcAtAtGtttAtAAttGAAActcGA -AAAAAAAAAAAAAAAA
SPA1-T01	pGGGtGAAtctAtGGGGtGGGcActGGGGtctc-AAAAAAAAAAAAAAAA
SPA1-T02	pGGGtGAAtctAtGGGGtGGGcActGGGGtc-AAAAAAAAAAAAAAAA
SPA1-T03	pGGGtGAAtctAtGGGGtGGGcActGGGG-AAAAAAAAAAAAAAAA
SPA1-T04	pGGtGAAtctAtGGGGtGGGcActGGGGtctcGA-AAAAAAAAAAAAAAAA
SPA1-T05	pGGGGAAtctAtGGGGtGGGcActGGGGtctcGA-AAAAAAAAAAAAAAAA
SPA1-T06	pGGGtAAtctAtGGGGtGGGcActGGGGtctcGA-AAAAAAAAAAAAAAAA
SPA1-T07	pGGGtGAtctAtGGGGtGGGcActGGGGtctcGA-AAAAAAAAAAAAAAAA
SPA1-T08	pGGGtGAActAtGGGGtGGGcActGGGGtctcGA-AAAAAAAAAAAAAAAA
SPA1-T09	pGGGtGAAttAtGGGGtGGGcActGGGGtctcGA-AAAAAAAAAAAAAAAA
SPA1-T10	pGGGtGAAtcAtGGGGtGGGcActGGGGtctcGA-AAAAAAAAAAAAAAAA
SPA1-T11	pGGGtGAAtcttGGGGtGGGcActGGGGtctcGA-AAAAAAAAAAAAAAAA
SPA1-T12	pGGGtGAAtctAGGGGtGGGcActGGGGtctcGA-AAAAAAAAAAAAAAAA
SPA1-T13	pGGGtGAAtctAtGGGtGGGcActGGGGtctcGA-AAAAAAAAAAAAAAAA
SPA1-T14	pGGGtGAAtctAtGGGGtGGcActGGGGtctcGA-AAAAAAAAAAAAAAAA
SPA1-T15	pGGGtGAAtctAtGGGGtGGGcActGGGtctcGA-AAAAAAAAAAAAAAAA
SPA1-T16	pGGGtGAAtctAtGGGGtGGGcAGGGG-AAAAAAAAAAAAAAAA
SPA1-M01	pGGGtGAAtctAtGGGGtGGGcAGGGG
SPA1-M02	GGGtGAAtctAtGGGGtGGGcAGGGG
SPA1-M03	pGGGtGAA(M)tctA(M)tGGG(M)G(M)tGGGcA(M)GGGG
SPA1-M04	pGGGtGAAtctA(M)tGGG(M)G(M)tG(M)G(M)G(M)cA(M)GGGG
SPA1-M05	pGGGtGAA(M)tctAtGGG(M)G(M)tG(M)G(M)G(M)cA(M)GGGG
SPA1-M06	pGGGtGAA(M)tctA(M)tGGGG(M)tG(M)G(M)G(M)cA(M)GGGG
SPA1-M07	pGGGtGAA(M)tctA(M)tGGG(M)G(M)tG(M)G(M)G(M)cAGGGG
SPA1-M08	pGGGtGAA(M)tctA(M)tGGG(M)G(M)tG(M)G(M)G(M)cA(M)GGGG
SPA1-M09	pGGGtGAA(M)tctA(M)U(M)GGG(M)G(M)tG(M)G(M)G(M)cA(M)GGGG
SPA1-M10	pGGGtGAA(M)tctA(M)tGGG(M)G(M)U(M)G(M)G(M)G(M)cA(M)GGGG
SPA1-M11	pGGGtGAA(M)tctA(M)tGGG(M)G(M)tG(M)G(M)G(M)C(M)A(M)GGGG
SPA1-M12	p^G^G^G^tGAA(M)tctA(M)tGGG(M)G(M)tG(M)G(M)G(M)cA(M)G^G^G^G
SPA1-M13	p^G^G^G^tG^A^A(M)tctA(M)tG(M)G^G(M)G(M)U(M)G(M)G(M)G(M)C(M)A(M)G^G^G^G
SPA1-M13-FAM	p^G^G^G^tG^A^A(M)tctA(M)tG(M)G^G(M)G(M)U(M)G(M)G(M)G(M)C(M)A(M)G^G^G^G-FAM
Scramble-FAM	pG^t^G^A(M)G^t^G(M)GcG(M)tG(M)U(M)G^AGtG(M)A(M)GGC(M)^G(M)^G^A(M)^G(M)-FAM
SPA1-M13-biotin	p^G^G^G^tG^A^A(M)tctA(M)tG(M)G^G(M)G(M)U(M)G(M)G(M)G(M)C(M)A(M)G^G^G^G-Biotin
**Modification List**	capital letter: RNA, small letter : DNA, p : Phosphate, N(F) : 2’-Fluoro-nucleotide, N(M) : 2’-O-methly-nucleotide, ^ : phosphorothiolate,

a

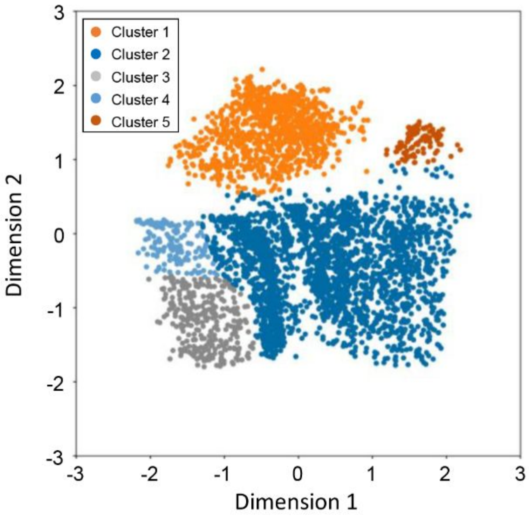

b

Cluster	ID	Sequence (5' to 3')
1	Sequence S1	GGGTAGTAATAATATAGTATATAGCGGGTCTCGA
2	Sequence S2	GGGTAGTAGTATTGTATTAGTGTATATATCTCGA
3	Sequence S3	GGGTATAGAACAATTGTTTAATAATGAAACTCGA
4	Sequence S4	GGGTAGTAGTAGTTTTATGTATAGTATATCTCGA
5	Sequence S5	GGGTATAGAACATATGTTTATAATTGAAACTCGA

c

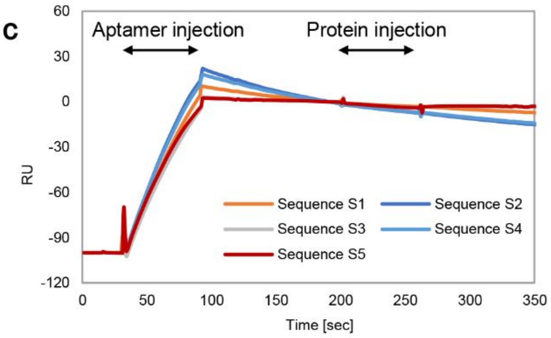

Figure S1. Candidate discovery using RaptGen. (a) Preprocessed sequencing data was subjected to RaptGen. After creating a latent space, representative sequences were selected as aptamer candidates. The plots indicate individual sequences in the sequencing data. The plots are indicated in the same color as the representative sequence. Candidate sequences were listed in the table (b). A and G are RNA and T and C are DNA. (c) Binding activity of candidate sequences were assessed by SPR experiment. PolyA tailed aptamers were generated by in vitro transcription. After aptamer immobilization on a sensor chip, 100 nM RBD protein was injected.

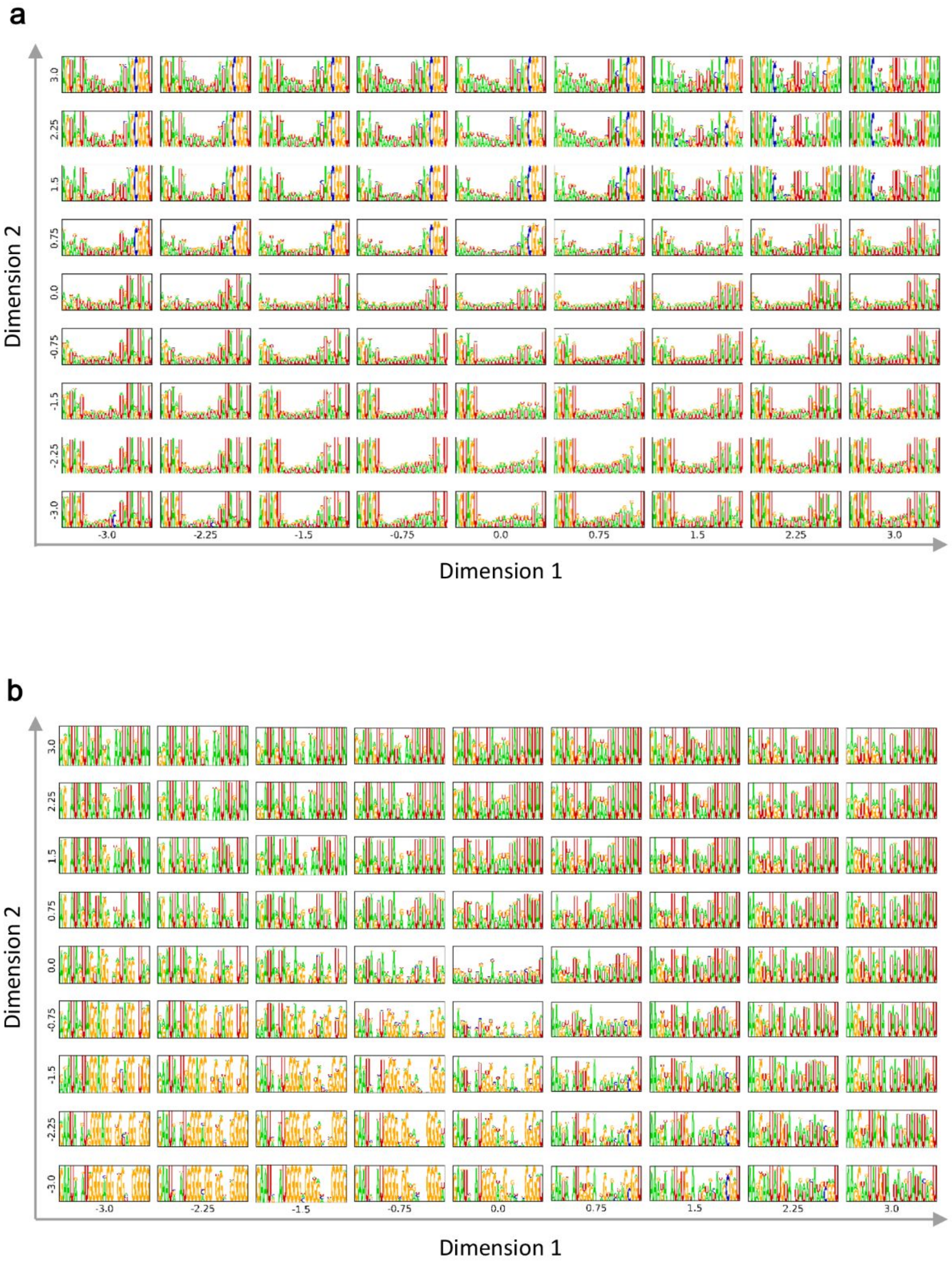

Figure S2. The sequence logo map for (a) 1st dataset and (b) 2nd dataset. The continuous motif indicated that the sequence in the data had the reference for specific subsequences.

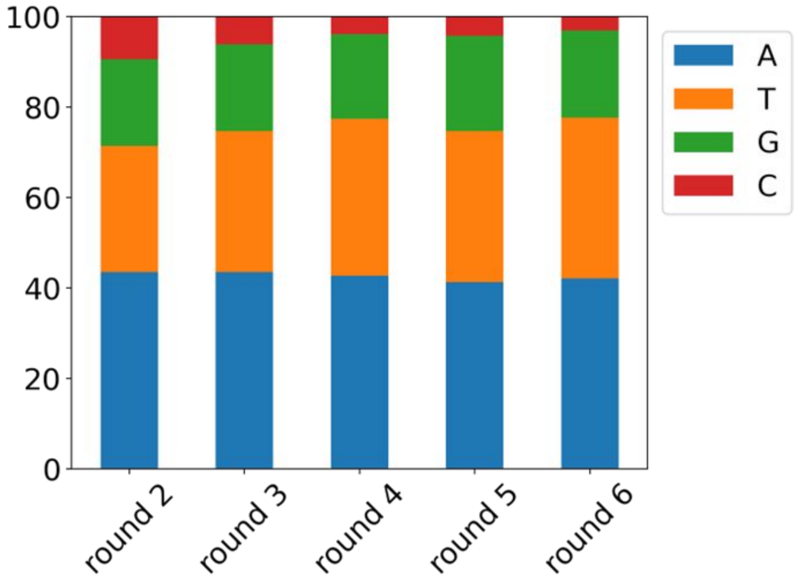

Figure S3. The proportion of each base in the enriched library.

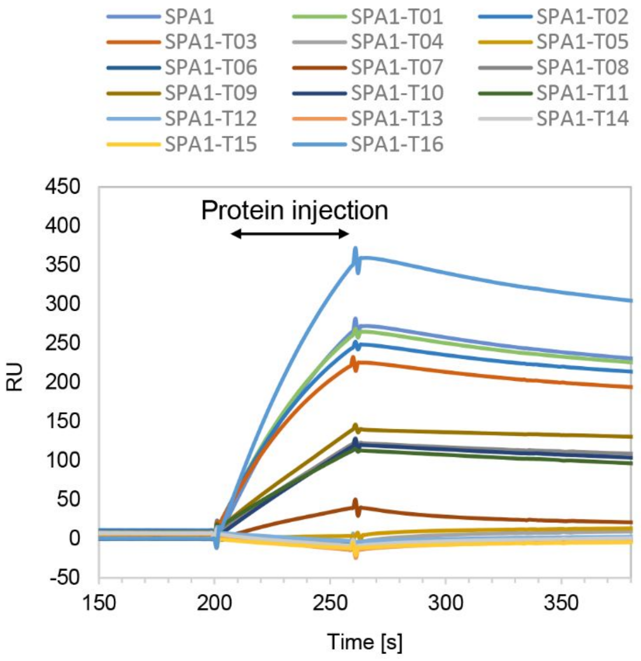

Figure S4. Binding activity of truncated aptamer(SPA1-T01 to -T16) and RBD of SARS-CoV-2 in SPR method. A Truncated aptamer having poly-$(rA)_{16}$ tail at 3'-end and its tails hybridized to poly-$(dT)_{16}$ immobilized on chip.

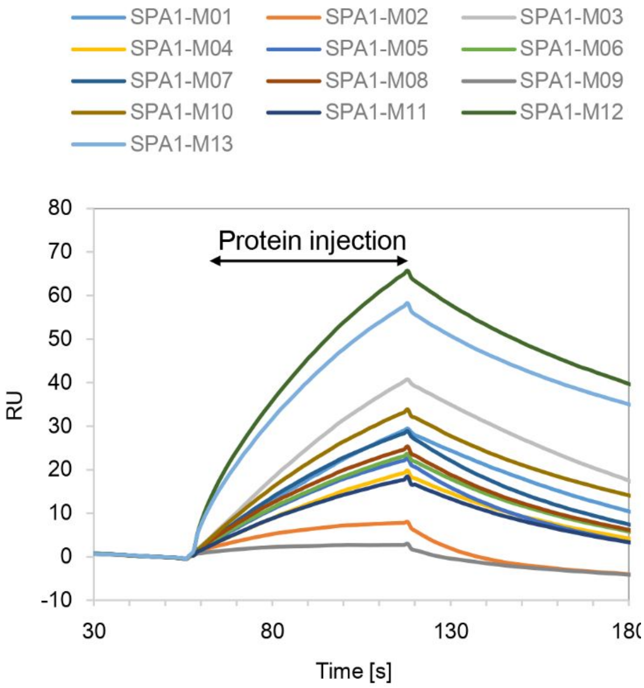

Figure S5. Binding activity of chemical-modified aptamer (SPA1-M01 to -M16) and RBD of SARS-CoV-2

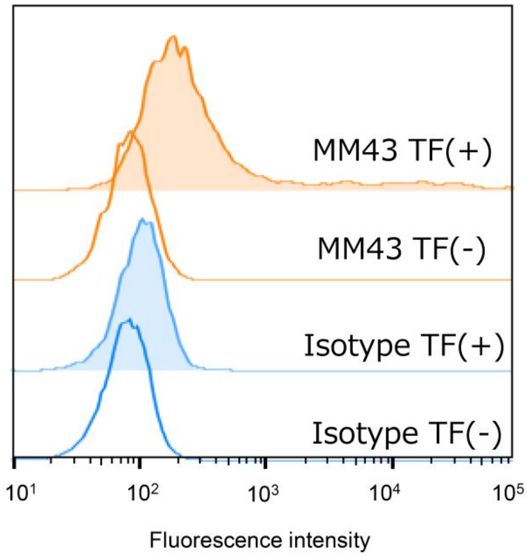

Figure S6. Flow cytometry to monitor the binding of MM43 antibody (orange) and Isotype control (blue) to plasmid transfected HEK293FT (TF+) and non-transfected HEK293FT(TF-).
